# Supplementary material for: Targeted Metabolomics Provide Chemotaxonomic Insights of Medicago ruthenica, with Coupled Transcriptomics Elucidating the Mechanism Underlying Floral Coloration
Source: Plants (Basel). 2022 Sep 15;11(18):2408. doi: 10.3390/plants11182408 (PMC9505912; doi:10.3390/plants11182408)
Supplement: Supplementary file 1 [file plants-11-02408-s001.zip › Supplementary Figures.pdf]

Figure S1

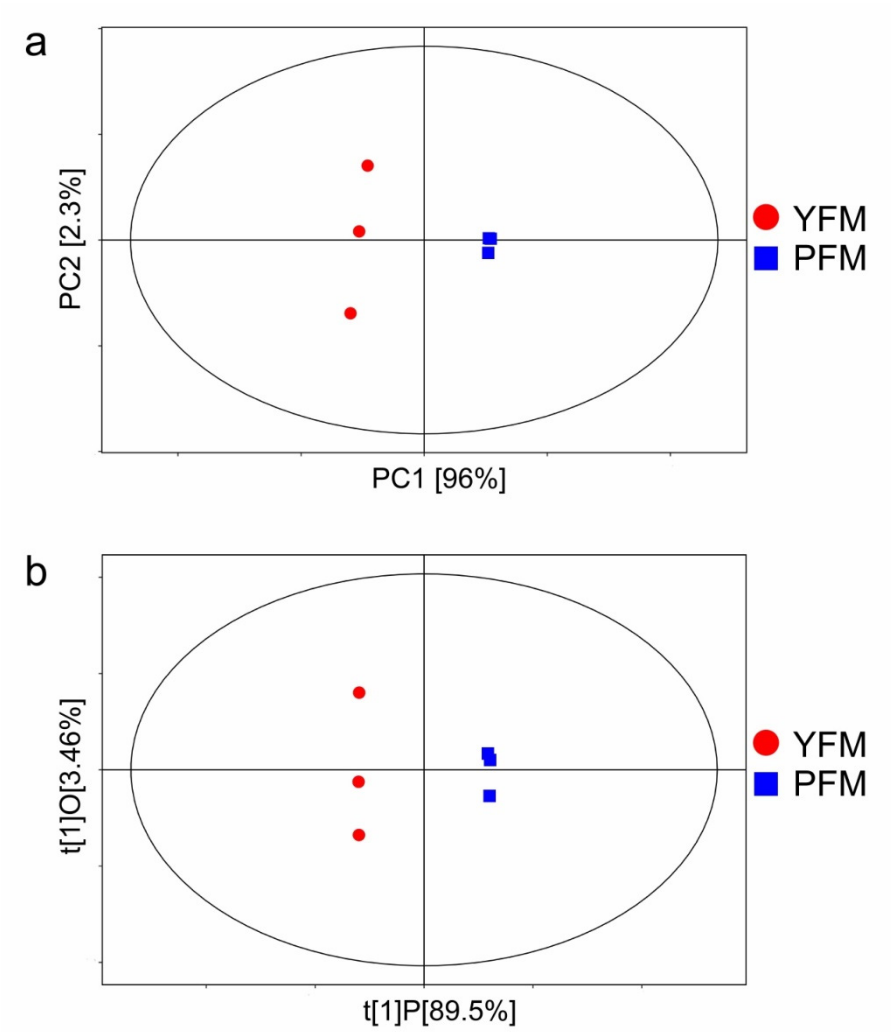

Figure S1. The preliminary statistics analysis (PCA, a) and orthogonal partial least-squares discrimination analysis (OPLS-DA, b) of metabolites. Petal samples were collected during flowering phase for targeted metabolomic. (**a**) Principal component analysis (PCA) used for understanding the relationships among the data matrix, the first principal component 1 (PC1) and the second principal component 2 (PC2) explained 96% and 2.3% of the components variability, respectively. (**b**) The orthogonal partial least-squares discrimination analysis (OPLS-DA) used for calculating the corresponding variable importance in projection (VIP) value, the OPLS-DA value was 89.5% indicate a complete separation of PFM and YFM, suggesting the significant difference of PFM and YFM in flavonoid metabolites. PCA and OPLS-DA were performed to evaluate differentially changed metabolites by SIMCA 16.0.2 software (Sartorious, Göttingen, Germany).

Figure S2

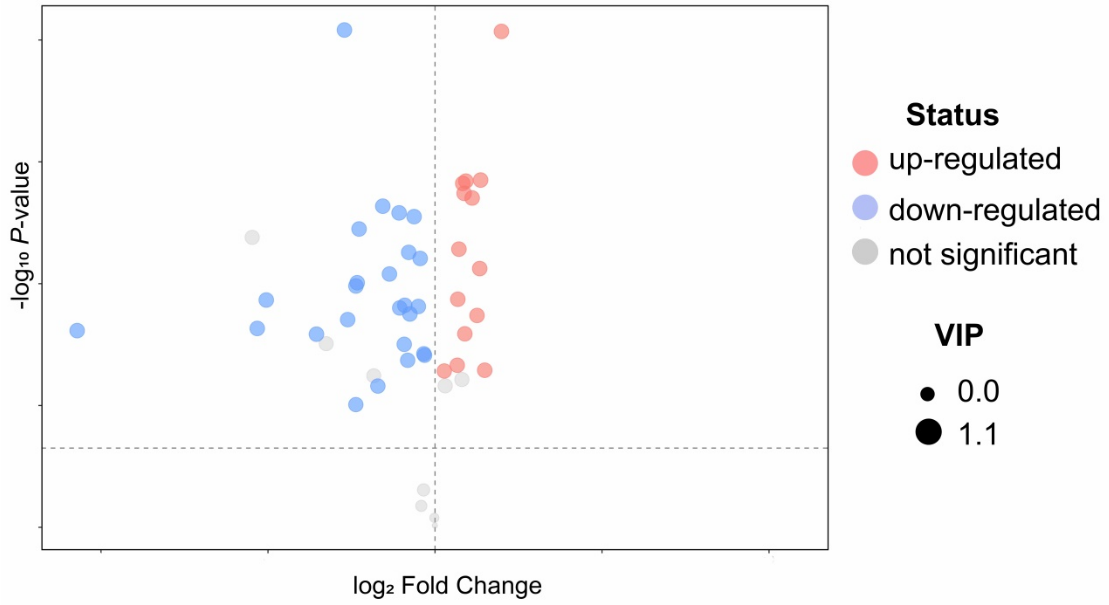

Figure S2. Volcano plot analysis. In this study, PFMs were served as a control group. Each point in the volcano map represented a kind of metabolite. Blue-colored circles represented down-regulated metabolites in YFM, red-colored circles represented up-regulated metabolites in YFM, and grey-colored circles represented there was not significant between PFM and YFM. The size of these circles represented variable importance in projection (VIP) value of these metabolites. In Fig S2, 14 metabolites were up regulated in YFM, 25 metabolites were down regulated in YFM and 9 metabolites with non-significant difference between YFM and PFM.
